# Impact of renal dysfunction on surgical outcomes in patients with aortic dissection

**DOI:** 10.1097/MD.0000000000015453

**Published:** 2019-05-17

**Authors:** Pei-Yi Fan, Chao-Yu Chen, Cheng-Chia Lee, Kuo-Sheng Liu, Victor Chien-Chia Wu, Pei-Chun Fan, Ming-Yang Chang, Jason Chih-Hsiang Chang, Ya-Chung Tian, Shao-Wei Chen

**Affiliations:** a Kidney Research Center, Department of Critical Care Nephrology, Division of Nephrology, Chang Gung Memorial Hospital, Linkou Branch; b Graduate Institute of Clinical Medical Science, College of Medicine, Chang Gung University; c Department of Cardiothoracic and Vascular Surgery, Chang Gung Memorial Hospital, Linkou Medical Center; d Department of Cardiology, Chang Gung Memorial Hospital, Linkou Medical Center, Taoyuan, Taiwan.

**Keywords:** prognosis, renal dysfunction, risk factors, type A aortic dissection (TAAD)

## Abstract

Preoperative renal dysfunction is associated with mortality in patients who undergo coronary artery bypass graft and valve surgery. However, the role of preoperative renal dysfunction in type A aortic dissection (TAAD) remains unclear. This study aimed to evaluate the impact of preoperative renal dysfunction on the outcome of surgical intervention in patients with TAAD.

We retrospectively studied the outcomes of 159 patients with TAAD who were treated at a tertiary referral hospital between 2005 and 2010. The demographics and surgical details of patients were analyzed according to their renal function. Risk factors for outcomes were analyzed using multivariable logistic regression. Thirty-two of the patients (20.1%) had preoperative serum creatinine of 1.5 mg/dL or more. The multivariable logistic regression model revealed independent risk factors of in-hospital mortality to be renal dysfunction (odds ratio [OR], 3.79; 95% confidence interval [CI], 1.64–8.77), preoperative shock (OR, 8.75; 95% CI, 2.83–27.02), and bypass time (OR, 1.008; 95% CI, 1.003–1.013). In addition, patients with renal dysfunction exhibited a lower 90-day survival rate than did patients without the condition (*P* of log-rank test = .005).

Preoperative renal dysfunction may have a critical role in the surgical outcomes of patients with TAAD. Additional large-scale investigations are warranted.

## 1 Introduction

The mortality rate of patients with acute unoperated type A aortic dissection (TAAD) increases 1% to 2% every hour within the first 2 days of an acute event. Thus, urgent surgical repair should be considered for all patients with TAAD. In-hospital mortality for patients managed surgically was found to be 27%, compared with 56% for those managed medically.^[1]^ Refractory pain, an age of over 70 years, and the absence of chest pain on admission have been reported as predictors of death. Aortic rupture was the most common cause of death identified in the International Registry of Acute Aortic Dissections (IRAD).^[2]^ In the past decade, neither the symptoms nor physical findings of TAAD have changed substantially. Advancements in diagnostic computed tomography (CT) have significantly reduced in-hospital mortality by facilitating early surgical intervention.^[3]^ Evidence suggests that acute kidney injury (AKI) following dissection worsens the prognosis and prolongs hospitalization.^[4,5]^ However, the potential effects of preoperative renal dysfunction have not been clearly explored in the literature. Recently, preoperative organ malperfusion was proved to affect the outcome in these patients.^[6]^ Therefore, our investigation evaluated the impact of preoperative renal dysfunction on mortality in patients with TAAD.

## 2 Methods

### 2.1 Study participants and design

The data of patients with TAAD treated between January 2005 and December 2010 were extracted for analysis from a prospectively collected database. This study design was approved by the institutional review board of Chang Gung Memorial Hospital (201601407B), and the need for individual consent was waived. We excluded patients who had undergone prior cardiac surgery, had end-stage renal disease, or died within 1 day after surgery. All patients had received contrast CT before surgery. The final cohort comprised 159 consecutive patients who received dissection repair in a single tertiary referral hospital. The patients were divided into 2 groups according to their serum creatinine (Cr) levels.

### 2.2 Data collection and definition

The baseline characteristics and demographic data of the patients were extracted from the database. The laboratory data comprised the preoperative values recorded on the operation date. Patient surgical details were also obtained from the database for analysis. Outcomes in terms of *de novo* dialysis, mortality, major complications, blood transfusions, and hospital stay were compared between the 2 renal function groups. Postoperative care was standardized in our intensive care unit (ICU) by 2 critical care specialists. Shock was defined as systolic pressure less than 90 mmHg.

### 2.3 Statistical analysis

Continuous variables were summarized as mean and standard deviation and were compared using the Student *t* test between the 2 groups defined according to preoperative Cr (<1.5 vs ≥1.5). The distribution of categorical variables in each group was compared using Fisher exact test. Associations of characteristics and operative conditions with risk of in-hospital mortality were assessed using univariate and multivariable logistic regression models. Variables with a *P* value less than .20 in the univariate models were subsequently introduced into the multivariable model with a stepwise selection procedure. Finally, Kaplan–Meier survival curves of 90-day mortality were plotted together with the log-rank test to compare the 2 groups. All statistical tests were 2-tailed and a *P* value less than .05 was considered significant. Data were analyzed using SPSS 22.0 software (IBM SPSS, Armonk, NY: IBM Corp).

## 3 Results

### 3.1 Study population characteristics and surgical details

One hundred and fifty-nine patients were examined in this study. The mean age was 57.5 years (*SD* = 13.2 years), and 27.0% of the patients were female. Thirty-two of the patients (20.1%) had preoperative Cr of 1.5 mg/dL or more. The baseline preoperative demographic and clinical characteristics are listed in Table 1. The 2 groups did not differ significantly in their baseline characteristics, namely age, sex, diabetes mellitus, hypertension, silent stroke, cardiac ejection fraction, chronic obstructive pulmonary disease (COPD), chronic kidney disease (CKD) and cirrhosis. Renal artery involvement was found in 36.1% in our cohort. The patients with Cr less than 1.5 mg/dL were more likely to have renal artery involvement than the others (40.9% vs 16.1% *P* = .012, respectively). Laboratory data for hemoglobin, platelet count, and prothrombin time also did not differ significantly between the groups, with the exception of Cr level.

**Table 1:**
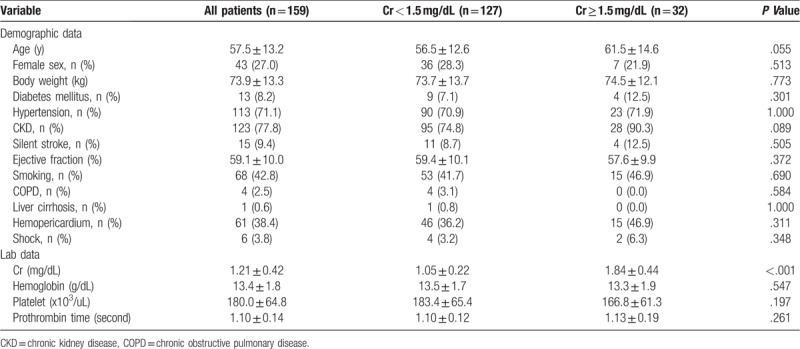
Preoperative demographic data and clinical characteristics.

The surgical details are shown in Table 2. They indicate that the patients with Cr less than 1.5 mg/dL were more likely to receive aortic arch repair (*P* = .043), which might reflect a more aggressive surgical strategy adopted for patients with a stable preoperative condition. No significant differences were present for additional coronary artery bypass graft, intraoperative bypass time, clamp time, arrest time, or brain protection strategy.

**Table 2:**
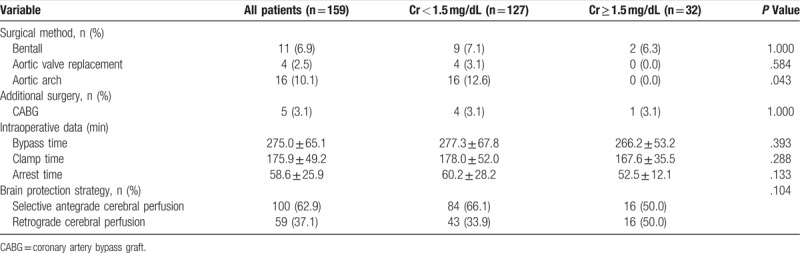
Operative and perioperative data.

### 3.2 Patient outcomes in different groups

In-hospital mortality was significantly higher in the group whose Cr was 1.5 mg/dL or more (34.4% vs 12.6%, *P* = .007). Other associated outcomes are listed in Table 3. However, no significant difference was observed in postoperative ventilator time, tracheostomy rate, ICU stay, hospital stay, unexpected bleeding check, or wound infection. In the first 48 hours after surgery, the transfusion amount did not differ significantly between the groups. The incidence of *de novo* dialysis in the patients with Cr of 1.5 mg/dL or more before surgery was also significantly higher than those with Cr of less than 1.5 mg/dL (18.8% vs 7.1%, *P* = .045).

**Table 3:**
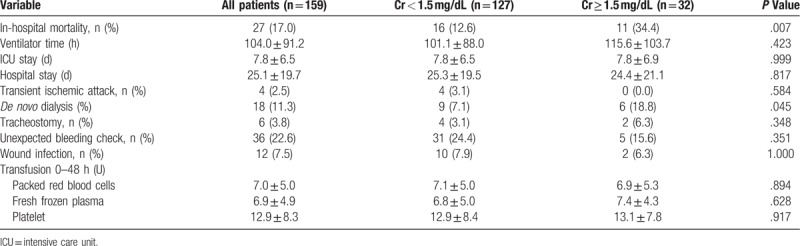
Postoperative outcomes.

### 3.3 Association between Cr and In-hospital mortality

**Figure 1:**
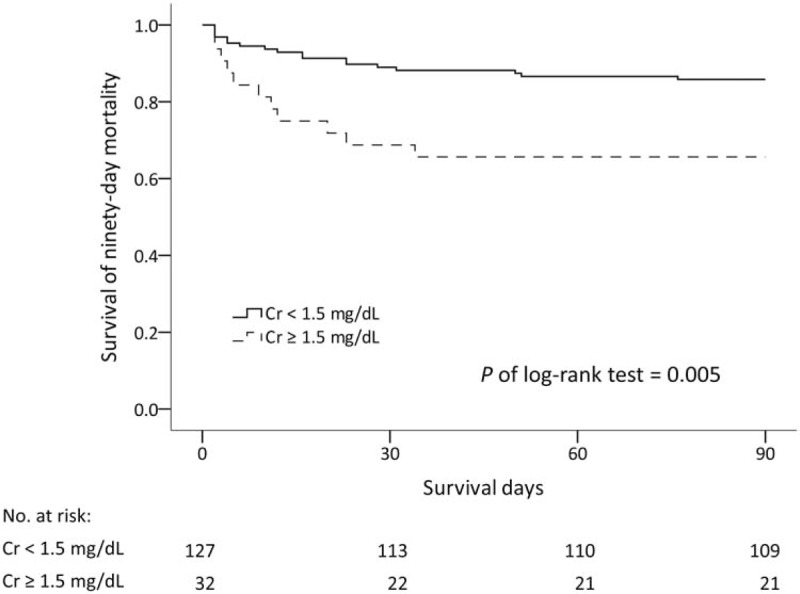
Kaplan–Meier survival curves for 90-day mortality according to the serum creatinine level.

To determine the risk factors for mortality, baseline characteristics and operative factors were analyzed using univariate logistic regression models; the results are shown in Table 4. The following variables were found to be significant: preoperative Cr of 1.5 mg/dL or more, preoperative shock, number of transfused platelets, prothrombin time, and intraoperative bypass time. The multivariable model indicated that preoperative renal function (odds ratio [OR], 3.79; 95% confidence interval [CI], 1.64–8.77), preoperative hypotension (OR, 8.75; 95% CI, 2.83–27.02), and bypass time (OR, 1.008; 95% CI, 1.003–1.013) were independently associated with risk of in-hospital mortality. Figure 1 presents the 90-day Kaplan–Meier survival curves for both groups, revealing that the patients with a higher Cr level had a lower 90-day survival rate (*P* of log-rank test = .005).

**Table 4:**
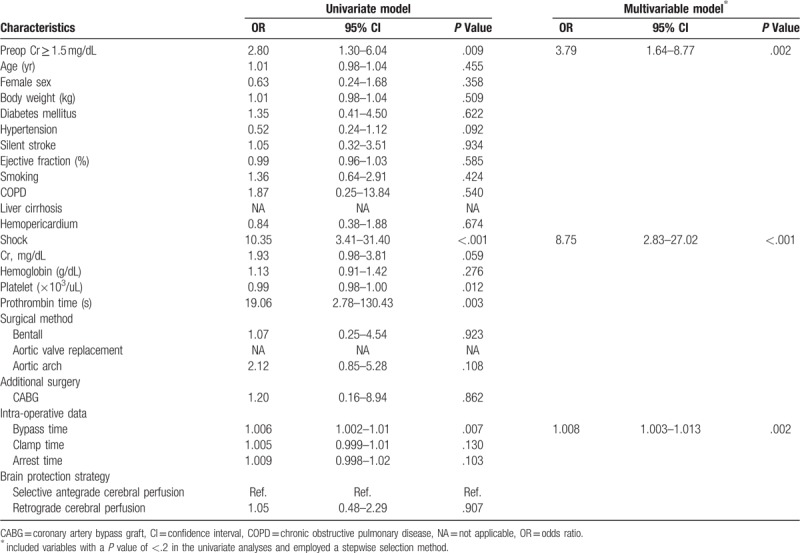
Associated factors of risk of in-hospital mortality.

## 4 Discussion

The relationship of renal dysfunction and surgery for aortic dissection have been discussed in many literatures. In most of these literatures, they investigated renal dysfunction as complication or postoperative outcome. A retrospective study by Kato et al. showed that TAAD is a risk factors for AKI after aortic dissection, and AKI increased the all-cause mortality.^[7]^ Another study by Pisimisis et al showed that thoracic aortic endograft has a significant rate of renal dysfunction.^[8]^ Some literatures revealed renal ischemia as risk factor of surgical outcome of acute TAAD. A retrospective review by Kazui et al demonstrated that renal ischemia is one of preoperative dissection-related complications and comorbidities, which significantly affect early and late survival rates after surgical treatment of acute TAAD.^[9]^ Our study focuses on the impact of pre-operative renal function to surgical outcome of patient with aortic dissection, which has not been investigated thoroughly. In the present study, we identified preoperative Cr level as an independent predictor for in-hospital mortality. The observed mortality rate of 17.0% is similar to those reported in prior publications.^[4,10,11]^ Using IRAD, Mehta et al examined 547 TAAD patients with surgical or medical intervention between 1996 and 1999 and found overall mortality of 32.5%.^[10]^ Nonsurvivors had a higher rate of acute renal failure during hospitalization than survivors did (11.3% vs 2.9%). Being aged 70 years or older, abrupt onset of chest pain, hypotension, kidney failure, pulse deficit, and abnormal ECG findings were independent risk factors for mortality. Conzelmann et al analyzed German Registry for Acute Aortic Dissection Type A data from 2137 surgical patients treated between July 2006 and June 2010 and found 30-day mortality of 16.9%.^[12]^ The preoperative and intraoperative factors were analyzed separately, revealing that preoperative factors such as age, coma, cardiopulmonary resuscitation, and number of malperfused organs were associated with mortality. The risk of mortality also increased with operation duration. Czerny et al investigated the same database and determined that organ malperfusion was a risk factor for death, with the mortality rate increasing with the number of malperfused organ.^[6]^ Neither arterial cannulation site for extracorporeal circulation, operative technique, nor arch intervention significantly affected 30-day mortality, which is the same to our results.

Perioperative renal dysfunction is associated with mortality and complications in coronary artery bypass surgery and valve repair.^[13–17]^ Tsai et al examined the data of 268 patients treated in a 6-year period and identified post-surgical AKI as a predictor of mortality.^[18]^ Another cohort of 117 patients with acute TAAD treated between 1997 and 2012 was found by Imasaka et al to have a surgical mortality rate of 14.9%.^[19]^ The Imasaka et al study calculated preoperative estimated glomerular filtration rate (eGFR) however, due to the small number of patients investigated, eGFR was not found to be an independent risk factor for surgical outcome. Our study is the first report to provide evidence that a preoperative Cr of 1.5 mg/dL or more is associated with a significant increase in adverse outcomes, including mortality.

There are several possible explanations of renal dysfunction in these patients. First, glomerular sclerosis resulting from chronic hypertension might be common and lead to CKD in patients with TAAD. Hagiwara et al examined 350 patients with TAAD who underwent surgery between 1988 and 1999 and found that 25.7% had CKD at the diagnosis of aortic aneurysm. In that cohort, only 160 patients received surgical intervention; 27.5% developed AKI (defined as a 150% increase in Cr). The proportion of CKD in patients who received surgery was unavailable and factors for in-hospital mortality were not analyzed. Comorbidities with atherosclerosis, older age, pulmonary emphysema, high blood pressure, and angiotensin II usually appear in patients with aortic dissection.^[20]^ Hypertensive emergency being present with dissection might also damage the kidney. Moreover, Onitsuka et al reported that obstruction of renal blood flow occurred in approximately 7% of cases involving acute dissection.^[21]^ They stated that reduced renal blood flow due to stenosis of the true lumen, which was oppressed by the false lumen, was associated with AKI. Furthermore, massive bleeding from a rupture or a myocardial infarction also causes renal dysfunction by alteration of hemodynamic stability. Although not suggested previously in the literature, contrast nephropathy might contribute to perioperative renal impairment. Cr is a delay marker of AKI and usually raised after 48 hours after kidney insult and our result showed that patients with Cr more than 1.5 mg/dL have more CKD. The perioperative Cr might reflect a mixture result of AKI and CKD. However, our data showed that the rate of *de novo* dialysis was higher in patients with preoperative renal dysfunction, suggesting the presence of AKI as well as contributing to mortality. A recently developed medication—dexmedetomidine, a selective a2 adrenoreceptor agonist—has a potential renoprotective effect for patients undergoing valvular heart surgery.^[22]^ Further study verifying the efficacy of dexmedetomidine in patients with TAAD is suggested in order to assess the contribution to the outcome.

### 4.1 Limitations

Despite the favorable results obtained in this study, several important limitations should be noted. First, this study was conducted at a single tertiary care medical center in Asia. Regional and ethnic differences should be considered, and the results may not be directly extrapolated to other patient populations. Second, the database used in this study lacked information such as preoperative symptons, dissection level, and renal artery involvement. Third, the relationship between shock and renal dysfunction is important. However, in the setting of our study, the patients presented with shock and renal dysfunction at the same time on arrival at our hospital. It is difficult to define which 1 originated the other precisely. Since aortic dissection is a disease needs emergent operation, the preoperative data were collected in limit time period. If we did not have baseline data of the patient's renal function, we could not distinguish AKI, CKD or AKI superimposed on CKD. We have made the efforts to distinguish AKI and CKD by the ways other than the level of creatinine. The patient diagnosed as aortic dissection did undergo CT for diagnosis. We have tried to differentiate acute or chronic renal dysfunction by the image of CT, such as measuring longitudinal diameter and cortical thickness and evaluating renal echogenicity and urinary tract status. However, different morphology of kidneys has been associated with different primary diseases. For example, Renal hypertrophy and glomerular hyperfiltration occur earlier in the course of diabetic nephropathy. Hence, we cannot define the how long the abnormal renal function exists. Last, the etiology of mortality after cardiac surgery is often multifactorial; thus, further application of the current findings for clinical usage should consider factors related to postoperative care.

## 5 Conclusion

Preoperative renal dysfunction is associated with mortality in patients with TAAD after surgical intervention. Additional large-scale investigations are warranted.

## Acknowledgments

This study was supported by grants from Chang Gung Memorial Hospital, Taiwan (CORPG5G0081 CORPG5G0071). Dr. C-H Chang was supported by the Ministry of Science and Technology (106-2314-B-182A-118 -MY3). The authors thank all the participating patients of the Kidney Research Center of Chang Gung Memorial Hospital, Linkou, Taiwan.

## Author contributions

**Conceptualization:** Ming-Yang Chang, Ya-Chung Tian, Shao-Wei Chen.

**Data curation:** Jason Chih-Hsiang Chang, Pei-Yi Fan, Chao-Yu Chen.

**Formal analysis:** Pei-Chun Fan, Jason Chih-Hsiang Chang, Pei-Yi Fan, Chao-Yu Chen.

**Investigation:** Victor Chien-Chia Wu, Ming-Yang Chang, Pei-Yi Fan, Chao-Yu Chen.

**Methodology:** Pei-Chun Fan, Ming-Yang Chang, Pei-Yi Fan, Chao-Yu Chen.

**Resources:** Shao-Wei Chen.

**Supervision:** Cheng-Chia Lee, Kuo-Sheng Liu, Jason Chih-Hsiang Chang, Ya-Chung Tian.

**Writing – original draft:** Pei-Yi Fan, Chao-Yu Chen.

**Writing – review & editing:** Chao-Yu Chen.
